# Development of an activity-directed selection system enabled significant improvement of the carboxylation efficiency of Rubisco

**DOI:** 10.1007/s13238-014-0072-x

**Published:** 2014-05-30

**Authors:** Zhen Cai, Guoxia Liu, Junli Zhang, Yin Li

**Affiliations:** 1 CAS Key Laboratory of Microbial Physiological and Metabolic Engineering, Institute of Microbiology, Chinese Academy of Sciences, Beijing, 100101 China; 2 Tianjin Institute of Industrial Biotechnology, Chinese Academy of Sciences, Tianjin, 300308 China

**Keywords:** carboxylation efficiency, CO_2_ fixation, directed evolution, Rubisco, *Synechococcus* sp. PCC7002

## Abstract

Photosynthetic CO_2_ fixation is the ultimate source of organic carbon on earth and thus is essential for crop production and carbon sequestration. Ribulose-1,5-bisphosphate carboxylase/oxygenase (Rubisco) catalyzes the first step of photosynthetic CO_2_ fixation. However, the extreme low carboxylation efficiency of Rubisco makes it the most attractive target for improving photosynthetic efficiency. Extensive studies have focused on re-engineering a more efficient enzyme, but the effort has been impeded by the limited understanding of its structure-function relationships and the lack of an efficient selection system towards its activity. To address the unsuccessful molecular engineering of Rubisco, we developed an *Escherichia coli*-based activity-directed selection system which links the growth of host cell solely to the Rubisco activity therein. A *Synechococcus* sp. PCC7002 Rubisco mutant with E49V and D82G substitutions in the small subunit was selected from a total of 15,000 mutants by one round of evolution. This mutant showed an 85% increase in specific carboxylation activity and a 45% improvement in catalytic efficiency towards CO_2_. The small-subunit E49V mutation was speculated to influence holoenzyme catalysis through interaction with the large-subunit Q225. This interaction is conserved among various Rubisco from higher plants and *Chlamydomonas reinhardtii*. Knowledge of these might provide clues for engineering Rubisco from higher plants, with the potential of increasing the crop yield.

**Electronic supplementary material:**

The online version of this article (doi:10.1007/s13238-014-0072-x) contains supplementary material, which is available to authorized users.

## Introduction

Rubisco (ribulose-1,5-bisphosphate carboxylase/oxygenase), the most abundant protein on earth, catalyzes the first step of photosynthetic CO_2_ fixation through the Calvin-Benson-Bassham cycle. This reaction directs the atmospheric inorganic carbon to the organic carbohydrate of life, and thus plays a pivotal role in crop production and global carbon cycle (Bracher et al., 2011; Field et al., 1998; Parry et al., 2013). Despite its biological importance, this step is greatly limited by the inefficient Rubisco kinetics owing to its low carboxylation activity and the competing oxygenase activity. The turnover numbers toward CO_2_ ($k_{\text{cat}}^{\text{C}}$) for Rubisco from various bacteria, algae and plants are in the range of 0.3–13.4 s^−1^ (Tcherkez et al., 2006; Whitney et al., 2011), which are several orders of magnitude lower than many common enzymes. Moreover, the abundant atmospheric O_2_ also competes the active sites of Rubisco, leading to the CO_2_-releasing and energy-consuming photorespiration process.

The contrast between its important role and poor capability has made Rubisco a primary engineering target to improve the biomass yield of the major food grain crops (Spreitzer and Salvucci, 2002; Zhu et al., 2010). Rubisco from higher plants is composed of eight large subunits (L, 50–55 kDa) and eight small subunits (S, 12–18 kDa), with the active sites only in the large subunit. Rational engineering of Rubisco mainly focused on the residues in or near the active sites (Hartman and Harpel, 1994; Marcus et al., 2003; Moreno and Spreitzer, 1999), the mobile loop 6 and the C-terminal strand of the large subunit which are involved in the conformational change during catalysis (Karkehabadi et al., 2007; Madgwick et al., 1998; Pearce and Andrews, 2003; Satagopan and Spreitzer, 2004; Spreitzer and Salvucci, 2002), the interface between the large and small subunits (Du and Spreitzer, 2000; Hong and Spreitzer, 1997; Shikanai et al., 1996), the structurally divergent βA-βB loop of the small subunit (Karkehabadi et al., 2005; Spreitzer et al., 2001; Spreitzer et al., 2005), and replacement of the whole small subunit (Genkov et al., 2010; Getzoff et al., 1998; Ishikawa et al., 2011). Mutagenesis of these regions always generated activity- and/or specificity-compromised Rubisco mutants. Only slight improvement in the carboxylation catalytic efficiency ($k_{\text{cat}}^{\text{C}}/K_{\text{M}}^{\text{C}}$, 20%–37%) (Genkov et al., 2006; Ishikawa et al., 2011; Madgwick et al., 1998) and CO_2_/O_2_ specificity ($k_{\text{cat}}^{\text{C}}K_{\text{M}}^{\text{O}}/K_{\text{M}}^{\text{C}}k_{\text{cat}}^{\text{O}}$, 5%–13%) (Genkov et al., 2006; Genkov et al., 2010; Madgwick et al., 1998; Spreitzer et al., 2005) were achieved. The limited success in rational engineering of Rubisco can be ascribed to our insufficient understanding on its intrinsic structure-function relationships.

Protein directed evolution based on random mutagenesis and selection offers an alternative strategy to engineer Rubisco. Recently a selection system using the phosphoribulokinase (PRK)-expressing *E. coli* has been reported (Greene et al., 2007; Mueller-Cajar et al., 2007; Mueller-Cajar and Whitney, 2008; Parikh et al., 2006). Theoretically, the accumulation of the catalytic product of PRK, ribulose-1,5-bisphosphate, is toxic to *E. coli* since it cannot be metabolized. Rubisco mutants which efficiently convert this dead-end product can restore cell growth and be selected. However, most of the selected mutants did not exhibit higher carboxylation activities except the large-subunit M259T mutation of Rubisco from *Synechococcus* PCC6301 which showed a 28% increase in the carboxylation catalytic efficiency (Greene et al., 2007). On the other hand, all the mutants showed 4- to 7-fold improved functional expression in *E. coli*.

The exploratory studies have shown the feasibility and utility of directed evolution in engineering the complex and promiscuous Rubisco. However, they appeared to favor the selection of expression-improved mutants rather than the activity-improved ones. It has been reported that very little (<2%) wildtype cyanobacterial Rubisco could correctly assemble into functional enzymes in *E. coli* (Mueller-Cajar and Whitney, 2008). We thus supposed that the current biased selection system was the consequence of the extremely low expression level of Rubisco therein. Under such circumstances, it might be much easier to improve the expression than the activity by several amino acids substitutions.

In this study, we addressed the unsuccessful molecular engineering of Rubisco by overcoming the bias problem of the current selection system. To minimize the possibility of selection of the undesired expression-improved mutants, the expression level of wildtype Rubisco was firstly maximized in *E. coli* through overexpressing the Rubisco-specific molecular chaperone and optimizing the expression conditions. When the functional expression of Rubisco in *E. coli* reached a “saturated” level, several point mutations could hardly improve its expression level further. Hence the activity-improved mutants could emerge. Based on this *E. coli*-based activity-directed selection system, we improved the specific carboxylation activity of Rubisco from *Synechococcus* sp. PCC7002 by 85%, which to our knowledge is the best improvement in engineering Rubisco. The deduced structure-function relationships from this Rubisco might be transplanted into Rubisco from higher plants since they share the same hexadecameric L_8_S_8_ structure.

## Results

### Saturation of the functional expression of Rubisco in *E. coli*

**Figure 1:**
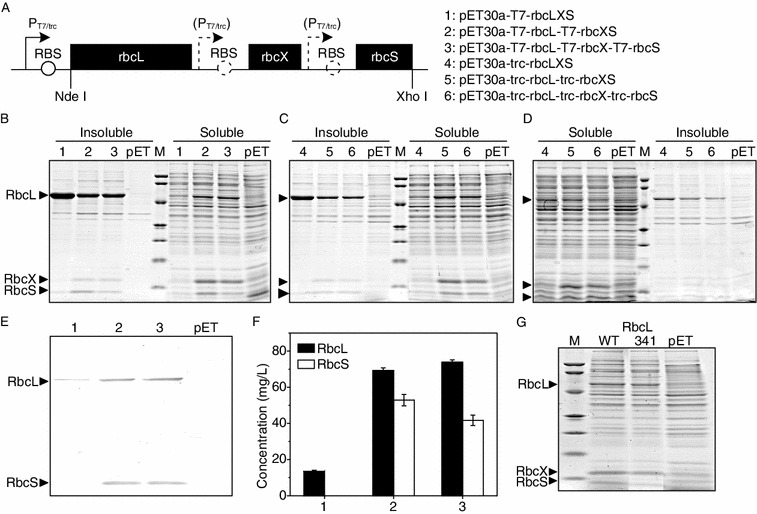
**Saturation of Rubisco expression in*E. coli***. (A) Six expression plasmids were constructed by inserting the chromosomal Rubisco operon *rbcL*-*rbcX*-*rbcS* into pET30a and placing one or two or three T7/trc promoters upstream of each gene. The expression plasmids were transformed into BL21(DE3) (B), DH5α (C) and HB101 (D) and their soluble and insoluble Rubisco expression levels were determined. The strain containing the empty plasmid pET30a was expressed in parallel as a negative control. Molecular weight standards from top to bottom are 94, 66.2, 45, 33, 26, 20 and 14.4 kDa. (E) Western blot analysis of the soluble Rubisco in BL21(DE3) using the antibodies against this Rubisco (synthesized by Epitomics, Inc., Hangzhou, China). (F) The concentrations of soluble Rubisco expressed in BL21(DE3) were calculated from (E) using software VisionWorksLS. Error bars: standard deviations of three independent expression and blotting experiments. (G) Comparison of the soluble Rubisco expression of wildtype Rubisco and its RbcL341 mutant in BL21(DE3) harboring the expression plasmid 2

The chromosomal Rubisco genes from *Synechococcus* sp. PCC 7002 are consisted of the large subunit *rbcL*, the chaperone *rbcX*, and the small subunit *rubS* in the order of *rbcL*-*rbcX*-*rbcS*. We subcloned the entire operon into the *Nde*I/*Xho*I sites of pET30a (Fig. 1A). To maximize the functional expression of Rubisco, two strong promoters (T7 and trc) and three *E. coli* hosts (BL21(DE3), DH5α, and HB101) were chosen. Consistent with those reported (Greene et al., 2007; Mueller-Cajar and Whitney, 2008), cyanobacterial Rubisco were hardly expressed in *E. coli* (plasmids 1 and 4, Fig. 1B–D). More than 90% of the expressed RbcL were in the insoluble fraction for either promoter in the three hosts. The soluble RbcX and RbcS were almost undetectable by SDS-PAGE and only a slight amount of their insoluble forms were seen. These results indicated that RbcL has been transcribed but folded incorrectly, whereas RbcX and RbcS might not achieve sufficient transcription from the upstream promoter located 1.5-kb away.

To further improve Rubisco expression, we designed to reduce RbcL misfolding and increase RbcS transcription. It has been reported that the Rubisco-specific chaperone RbcX could bind the disordered C-terminal region of RbcL, act as a molecular staple in stabilizing the RbcL dimmers, and facilitate its folding and assembly (Liu et al., 2010). Thus an additional promoter (T7 or trc) containing the RBS site was fused in the upstream of the *rbcX* gene to overexpress this chaperone protein for the correct RbcL folding. To increase RbcS transcription, a third T7(trc)-RBS was inserted at the upstream of *rbcS*.

As expected, the dramatic increase of soluble RbcL as well as the decrease of its insoluble fraction were seen for the three hosts after RbcX overexpression (plasmids 2 and 5 compared with 1 and 4, respectively, Fig. 1B–D). Take BL21(DE3) as an example, quantitative analysis of the immunobloted soluble protein revealed that there was a 5.1-fold increase for soluble RbcL after RbcX overexpression (plasmids 2 compared with 1, Fig. 1E and 1F). Moreover, both soluble and insoluble RbcS were simultaneously increased after addition of the second promoter in the three hosts (plasmids 2 and 5 compared with 1 and 4, respectively, Fig. 1B–D), which might be the consequence of a closer promoter. However, the addition of a third promoter at the upstream of *rbcS* reduced soluble RbcS by 21% (plasmids 3 compared with 2, Fig. 1F), suggesting that the RbcS expression was no longer limited by its transcription. Finally BL21(DE3) containing the expression plasmid 2 (with two T7 promoters each at the upstream of *rbcL* and *rbcX*) was chosen as the best expression system for Rubisco. Notably, the soluble Rubisco expressed in this system was several folds higher than that in the previous selection system (Mueller-Cajar et al., 2007; Mueller-Cajar and Whitney, 2008) which employed trc promoter in HB101 without chaperon overexpression (plasmid 2 in Fig. 1B compared with plasmid 4 in Fig. 1D).

To confirm that Rubisco expression in our BL21(DE3)/plasmid 2 expression system was saturated, the reported best expression-improved mutant was constructed and its expression level in our expression system was examined. The large-subunit F341I mutation of Rubisco from *Synechococcus* PCC7002 shows an 11-fold improvement in its functional RbcL expression in XL1-Blue (Mueller-Cajar and Whitney, 2008). However, in our expression system the soluble expression of RbcL341 mutant was a little lower than the wildtype (Fig. 1G), confirming the saturation of Rubisco soluble expression in our expression system.

### Construction of an activity-directed selection system for Rubisco

**Figure 2:**
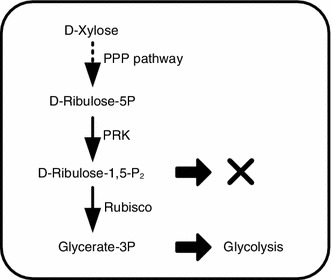
**Selection rationale**. D-Xylose is converted to D-ribulose-5-phosphate by the pentose phosphate pathway (PPP) in *E. coli*. Expression of PRK produces D-ribulose-1,5-bisphosphate which cannot be metabolized and then causes growth arrest. Co-expression of Rubisco converts this dead-end product to glycerate-3-phosphate, an intermediate during glycolysis, and then restores cell growth

The activity-directed selection system for Rubisco was constructed by linking the growth of host cell solely to its intracellular Rubisco activity. To establish such a linkage, we adopted the selection rationale of “PRK poisoning followed by Rubisco rescuing” (Fig. 2), but implemented it based on the saturated Rubisco expression to avoid the selection of expression-improved mutants.

**Figure 3:**
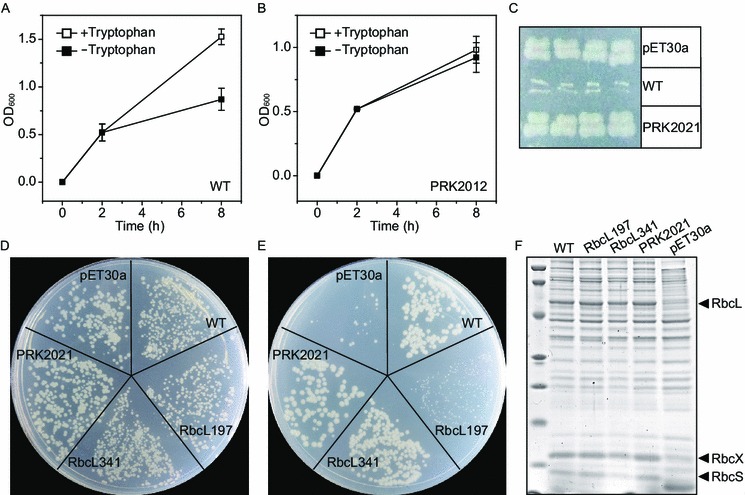
**Construction of the activity-directed selection system for Rubisco**. In all the panels, WT represents the selection plasmid containing the wildtype *rbc* and *prk* genes. RbcL197, RbcL341 and PRK2021 are the plasmids containing the corresponding Rubisco and PRK mutants. BL21(DE3) cells transformed with the wildtype selection plasmid (A) and its inactive PRK2021 mutant (B) were cultured in M9 minimal medium containing 50 μg/mL kanamycin, 1% (*w*/*v*) xylose, 0.05% (*w*/*v*) casamino acids, and 50 μg/mL tryptophan at 37°C at zero point. When the OD_600_ reached 0.5, the culture was divided into two halves. One half served as control, with continuous shaking in the tryptophan-containing medium for 6 h at 22°C (open squares). The other half was centrifuged, resuspended in the same medium without tryptophan for PRK expression, and shaken in parallel (solid squares). Error bars: standard deviations of three independent expression experiments. (C) BL21(DE3) cells harboring the wildtype and PRK2021 plasmids were streaked on M9 minimal plate containing 50 μg/mL kanamycin, 1% (*w*/*v*) xylose, 0.05% (*w*/*v*) casamino acids for PRK expression. BL21(DE3) cells transformed with different plasmids were plated on a non-selective (D) and a selective plate (E). The non-selective plate was M9 minimal agar plate containing 50 μg/mL kanamycin, 1% (*w*/*v*) xylose, 0.05% (*w*/*v*) casamino acids, and 50 μg/mL tryptophan, on which both Rubisco and PRK were not expressed. The selective plate was prepared by subtracting tryptophan and adding 0.02 mmol/L IPTG to the non-selective plate for both genes expression. The non-selective plate was incubated at atmospheric CO_2_ for 3–4 days at room temperature, while the selective plate was incubated at the same condition except for placing in the chamber filled with 2.5% (*v*/*v*) CO_2_. (F) Soluble Rubisco expression of various Rubisco and PRK mutants in our selection system

To construct such an activity-directed selection system, the *prk* gene from *Synechococcuselongatus* PCC7942 together with a tryptophan-regulated promoter *trpR*-P_trp_ were inserted into the backbone of the expression plasmid 2 to generate the selection plasmid. After transformation of the selection plasmid into BL21(DE3), inhibited cell growth both on liquid and solid media was observed upon tryptophan removal for PRK expression (Fig. 3A and 3C). Additionally, an inactive PRK mutant PRK2021, which carries K20M and S21A mutations in the conserved nucleotide-binding sites of ATP-binding proteins (Higgins et al., 1986), was constructed to replace the wildtype gene in the selective plasmid. This inactive PRK mutant showed no difference in growth rate after tryptophan removal (Fig. 3B and 3C), further proving the “poison” function of PRK.

The “rescue” function of Rubisco was similarly demonstrated by constructing its inactive mutant RbcL197 and comparing its performance with the wildtype enzyme. The RbcL197 mutant codes a K197M mutation in RbcL, which destroys the binding of nonsubstrate CO_2_ to form a carbamate and thus prevents Mg^2+^ binding and active-site activation (Cleland et al., 1998). This single mutation did not affect the soluble expression level of Rubisco (Fig. 3F). Hence the observed phenotype difference was only the consequence of their different activities. Under the non-selective condition (i.e., without induction of PRK and Rubisco), cells carrying the wildtype selection plasmid and its RbcL197 variant exhibited similar growth (Fig. 3D). When cells were spread on the selective plate containing 0.02 mmol/L IPTG and subtracting tryptophan for both Rubisco and PRK expression, wildtype Rubisco exhibited normal cell growth, while the inactive RbcL197 mutant still suffered the growth arrest (Fig. 3E), suggesting that it was indeed the Rubisco activity that rescued the cell.

As mentioned above, the reported selection systems preferred to select the expression-improved Rubisco mutants and thus failed to improve its activity. For example, the frequently selected large-subunit F345I mutant of *Synechococcus* PCC6301 Rubisco showed a 7-fold improvement in the functional expression in *E. coli* but a 17% decrease in its carboxylation catalytic efficiency (Mueller-Cajar and Whitney, 2008). To test whether our selection system suffers this bias problem, the reported best expression-improved mutant (RbcL341) was examined in our system. Compared with the wildtype Rubisco, RbcL341 did not show better growth and would not be selected by our selection system (Fig. 3E). In other words, comparison of the colony sizes of Rubisco mutants in our selection system enables selection of the activity-improved one (WT compared with RbcL197, Fig. 3E) and deselection of the expression-improved one (RbcL341 compared with WT, Fig. 3E), demonstrating that it is an activity-directed selection system. In addition, the selection time for colony visualization on the selective plate was dramatically reduced from ten days in the reported selection system (Mueller-Cajar et al., 2007; Mueller-Cajar and Whitney, 2008) to three to four days in our system, which might be the consequence of the increased metabolic flux of the PRK-Rubisco shunt by the saturated Rubisco expression.

### Directed evolution of *Synechococcus* PCC 7002 Rubisco

**Figure 4:**
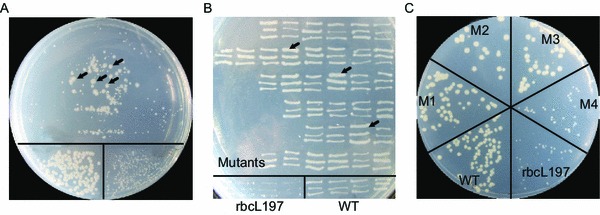
**Stepwise selection scheme**. (A) BL21(DE3) cells transformed with the Rubisco mutants library (upper region), the wildtype Rubisco (WT, lower left region) and the inactive Rubisco (RbcL197, lower right region) were plated on the selective plates for pre-screening. Preparation and incubation of the plates were described in Fig. 2. The colonies with bigger or similar size compared with the wildtype (indicated by arrows) were streaked on selective plates for re-screening. (B) The strains with better growth than the wildtype on the selective plates (indicated by arrows) were picked for a double-check. (C) Plasmids were extracted and transformed into the fresh BL21(DE3) cells and then incubated under the selective condition. The wildtype and inactive RbcL197 were prepared in parallel for comparison. The bigger colonies (e.g., M2 and M3 in this plate) were chosen for activity assays of the crude cell extracts

Random mutations were simultaneously introduced into the nonadjacent *rbcL* and *rbcS* genes whereas the middle T7-*rbcX* sequence was kept unaltered. The resulted Rubisco mutants contained an average of one to two amino acid substitutions for each of the large and small subunits. About 15,000 *E. coli* transformants harboring the Rubisco variants were directly spread onto the selective plates for pre-screening. Cells transformed with the wildtype Rubisco and the RbcL197 mutant were simultaneously spread to serve as positive and negative controls, respectively. After three to four days’ incubation at room temperature in an air-tight container (IPC250-T2, Lange Automation Technology Co., Baoding, China) filled with the air supplemented with 2.5% (*v/v*) CO_2_, colonies with various sizes were visualized for the cells transformed with the Rubisco mutants library (Fig. 4A), indicating possibly different activities of these mutants. A total of 500 large colonies, together with the wildtype and RbcL197 were picked and streaked on selective plates for re-screening (Fig. 4B). This time 74 mutants exhibited higher density than the wildtype. The plasmids in the 74 mutants were extracted and transformed into fresh BL21(DE3) cells for a double-check (Fig. 4C). Fifteen mutants with bigger colony sizes were subjected to the activity assay. The most active Rubisco mutant M6-5 was purified and its specific activities and kinetics were assayed (Table 1). The M6-5 mutant evinced an 85% increase in specific carboxylation activity and a 45% increase in catalytic efficiency towards CO_2_ ($k_{\text{cat}}^{\text{C}}/K_{\text{M}}^{\text{C}}$). The improved activity was the combinatory results of a significantly improved turnover number towards CO_2_ (120% increased $k_{\text{cat}}^{\text{C}}$), a decreased affinity towards CO_2_ (51% increased $K_{\text{M}}^{\text{C}}$) and a nearly unchanged affinity towards RuBP.

**Table 1:** Specific activities and kinetics parameters of the wildtype and evolved Rubisco

Rubisco	Specific carboxylation activity (U/mg)	$k_{\text{cat}}^{\text{C}}$ (s^−1^)	$K_{\text{M}}^{\text{C}}$ (μmol/L)	$k_{\text{cat}}^{\text{C}}/K_{\text{M}}^{\text{C}}$ [s^−1^ (mmol/L)^−1^]	$K_{\text{M}}^{\text{RuBP}}$ (μmol/L)
WT	0.91 ± 0.04	6.7 ± 0.6	131.5 ± 4.6	51	51.8 ± 8.4
M6-5	1.68 ± 0.14	14.6 ± 1.1	198.0 ± 12.4	74	49.4 ± 8.7

±: standard deviations of five to six independent experiments including expression, purification and activity assay

## Discussion

Rubisco has long been a target for improving photosynthetic carbon fixation, with the expectation that a more efficient enzyme would be beneficial for increasing the crop yield (Parry et al., 2013; Zhu et al., 2010). Numerous effort in rational design as well as directed evolution has been made, but limited success has been achieved in improving its carboxylation activity (Bracher et al., 2011; Stec, 2012; Whitney et al., 2011). The most remarkable improvement in engineering this important enzyme is its several-fold increased heterologous functional expression in *E. coli* (Greene et al., 2007; Mueller-Cajar and Whitney, 2008; Parikh et al., 2006). In this study, we blocked the evolutionary path of Rubisco towards improved expression via pre-saturating the expression by RbcX overexpression. Thereby the evolutionary force generated by mutagenesis was channeled to the path towards improved carboxylation activity. Based on this, an activity-directed *E. coli* selection system was constructed by linking the host growth to the Rubisco activity therein. As expected, a mutant with 85% improved specific carboxylation activity was selected. Rubisco appears to be extremely difficult to be engineered. Decades of engineering gains numerous activity-compromised Rubisco mutants. The best improvement in its catalytic efficiency towards CO_2_ ($k_{\text{cat}}^{\text{C}}/K_{\text{M}}^{\text{C}}$) reported to date is only 37% (Genkov et al., 2006). However, we improved the catalytic efficiency of Rubisco towards CO_2_ by 45% in one round of evolution, demonstrating the efficiency of this activity-directed selection system.

There is no doubt that the selection system demonstrated here is able to select the truly activity-improved Rubisco mutants. However, the current version of selection system appears to show a high false-positive rate. Possible explanations are shown below. Firstly, the colony size of cell growth on agar plate was somewhat related to its location. Usually the cells in the edge region grow bigger than those in the middle region. To avoid the miss of true positive ones, a loose cutoff of colony selection and a three-tier screening scheme were employed. Then all colonies with slightly bigger or similar sizes were selected for the next tier of screening. Secondly, we found that the plasmids in some big colonies only harbored the partially digested *prk* gene, which might be the consequence of some unknown errors during enzymatic digestion in library construction. These PRK-disrupting cells exhibited no growth arrest and were easily selected. For the two reasons, 500 large colonies were selected after pre-screening but finally only one positive colony was found. Since picking and streaking hundreds of colonies on agar plates could be performed routinely, and the errors during enzymatic digestion could be avoided through a meticulous quality control, we believe that those would not reduce the utility of this selection system.

The kinetic parameters of Rubisco from *Synechococcus* PCC7002 determined in this study were a little lower than those reported ($k_{\text{cat}}^{\text{C}}$ value of 13.4 s^−1^ and $K_{\text{M}}^{\text{C}}$ value of 246 μmol/L) (Andrews and Lorimer, 1985). This might be the variations of different assays. The literature adopted a radioisotopic assay with ^14^C-labbled CO_2_, while we used the spectrophotometric method with enzyme-coupled reactions. It is noteworthy that different kinetic properties for a certain Rubisco have been reported, even using the same radioisotopic assay. For example, the $k_{\text{cat}}^{\text{C}}$ and $K_{\text{M}}^{\text{C}}$ values of *Synechococcus* PCC6301 Rubisco were reported to be 11.6 s^−1^ and 340 μmol/L in one literature (Morell et al., 1994) and 4.9 s^−1^ and 186 μmol/L in another (Satagopan et al., 2009). Those values of *Synechococcus* PCC6803 Rubisco were 14.3 s^−1^ and 268 μmol/L in one literature (Marcus et al., 2011) and 9.1 s^−1^ and 180 μmol/L in another (Marcus et al., 2005). In this study, the mutant M6-5 and wildtype enzymes were assayed simultaneously and the values shown in Table 1 were the average results of five to six independent experiments including expression, purification and activity assay. Because M6-5 reproducibly showed elevated carboxylation activity in all the assays, the results were convincing.

**Figure 5:**
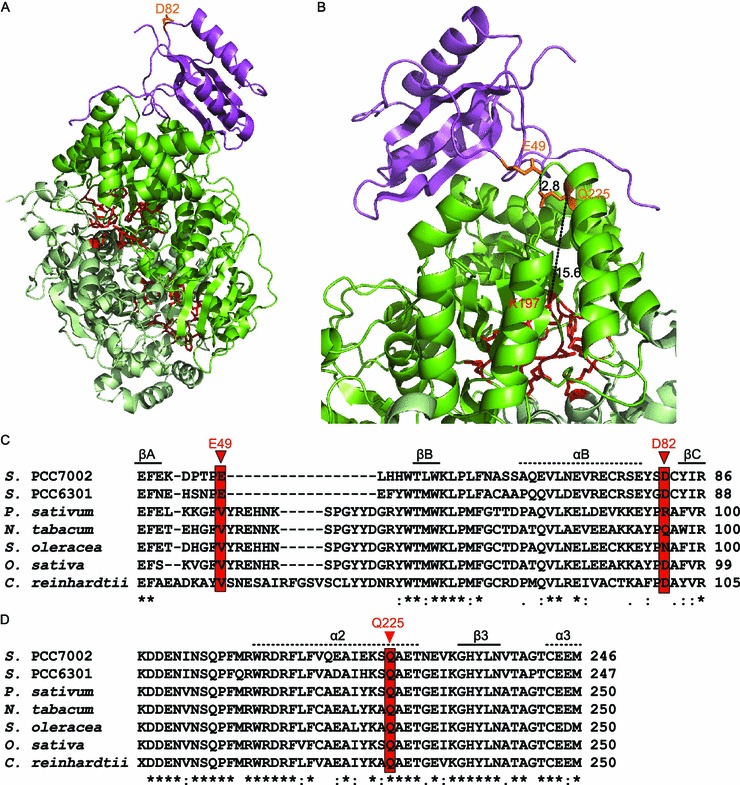
**Location and conservation of the two small-subunit mutations found in M6-5**. The hexadecameric structure of Rubisco from *Synechococcus* sp. PCC7002 was constructed by SWISS-MODEL using Rubisco from *Synechococcus* sp. PCC6301 (Protein Data Bank code: 1RBL) as a template. The sequence identities of the large and small subunits were 87% and 67%, respectively. One small subunit (magenta) and the adjacent large-subunit dimer (dark and light green) were shown. The conserved activity sites containing the large-subunit residues E56, T61, W62, N119, T169, K171, K173, K197, D199, E200, H290, R291, H323, K330, L331, S375, G376, G377, G399, and G400 were represented by red sticks. All the images were generated by PyMOL (version 0.99; DeLano Scientific, San Carlos, CA, USA). (A) Location of D82. (B) E49 is in close contact with large-subunit Q225. The number beside the black dash is the distance (in Å) between the two connected atoms. Sequence alignments of the small (C) and large (D) subunits of seven Rubisco enzymes based on their crystal structures. Protein Data Bank codes 1RBL, 4HHH, 4RUB, 8RUC, 3AXM, and 1GK8 were chosen for Rubisco from *Synechococcus elongatus* PCC6301, *Pisum sativum* (pea), *Nicotiana tabacum* (tobacco), *Spinacia oleracea* (spinach), *Oryza sativa* Japonica Group (rice), and *Chlamydomonas reinhardtii*. The conservation of amino acids were labeled according to ClustalX specification, with symbols “*”, “:”, and “.” representing identical, conserved, and semi-conserved residues

**Figure 6:**
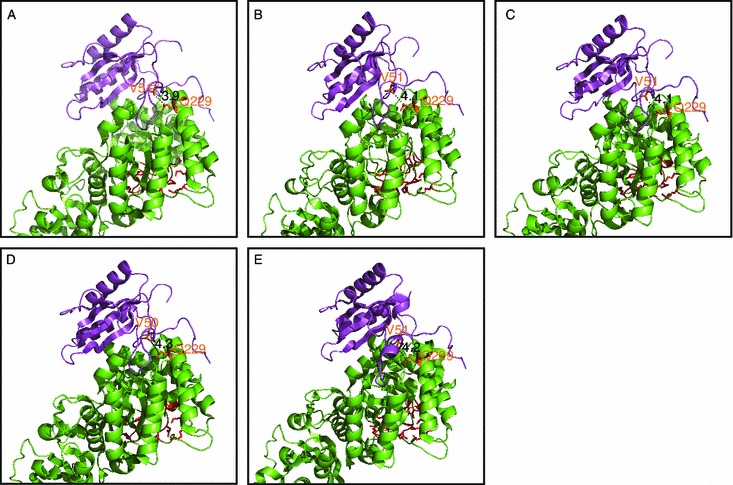
**Location and conservation of the small-subunit E49 and its contacted large-subunit Q225 in Rubisco from other species**. (A) *Pisum sativum* (pea, PDB code 4HHH), (B) *Nicotiana tabacum* (tobacco, PDB code 4RUB), (C) *Spinacia oleracea* (spinach, PDB code 8RUC), (D) *Oryza sativa* Japonica Group (rice, PDB code 3AXM), and (E) *Chlamydomonas reinhardtii* (PDB code 1GK8). One small subunit (magenta) and its adjacent large-subunit (green) were shown. The conserved activity sites containing the large-subunit residues E60, T65, W66, N123, T173, K175, K177, K201, D203, E204, H294, R295, H327, K334, L335, S379, G380, G381, G403, and G404 were represented by red sticks. The small-subunit V51/50 marked by orange sticks in the five Rubisco structures correspond to E49 in Rubisco from *Synechococcus* sp. PCC7002, while the marked large-subunit Q229 equal to Q225 in *Synechococcus* sp. PCC7002. The number beside the black dash is the distance (in Å) between the two connected atoms. All the images were generated by PyMOL (version 0.99; DeLano Scientific, San Carlos, CA, USA)

The best mutant M6-5 contains two mutations E49V and D82G in the small subunit. D82 is not in contact with the large subunit (Fig. 5A), making it difficult to explain its effect on Rubisco catalysis. E49 locates in the extensively studied βA-βB loop which enters the central solvent channel. The OE2 atom of E49 is closely associated with the OE1 atom of large-subunit Q225 (Fig. 5B). The hydrophilic glutamic acid at residue 49 changes to a much smaller hydrophobic valine in M6-5. This change might benefit the hydrophobic interaction between the small-subunit βA-βB loop and the large subunit, and thus facilitate the heterodimer interaction (van Lun et al., 2011). Interestingly, this E49 site is already valine in Rubisco from higher plants and *Chlamydomonas reinhardtii*, whereas the large-subunit Q225 is well conserved for all Rubisco (Fig. 5C and 5D). In these species, the corresponding small-subunit valine and large-subunit glutamic acid are also in contact with each other, with a distance of approximate 4 Å between the two side-chain atoms (Fig. 6). These results indicate that these two sites might be a conserved large/small-subunit interactive point among various Rubisco. Notably, Q225 which locates in the α-helix 2 of the α/β-barrel is at least 15 Å away from the nearest conserved active site residue K197 (Fig. 5B). How this distant mutation influences catalysis is dependent on the comparison of the crystal structures of the mutant and wildtype Rubisco, which is now under investigation of our group.

In summary, we provided an efficient approach for improving the carboxylation activity of Rubisco, one of the most abundant proteins on earth and also probably the most difficult one to be engineered. Our results provide new insights into the structure-function relationships of this important enzyme. This might advance our understanding and capacity to engineer Rubisco from higher plants, with the aim to improve the yield of major food crops.

## Materials and Methods

### Plasmids construction

The primers used were summarized in Table S1. *Synechococcus* sp. PCC7002 Rubisco genes were inserted into the *Nde*I/*Xho*I sites of pET30a, resulting plasmid 1. A second and a third T7 promoters containing the ribosomal binding site (RBS) region were fused in the upstream of *rbcX* and *rbcS*, yielding plasmids 2 and 3, respectively. The trc promoter was synthesized by primer annealing and inserted in the *Xba*I site of plasmid 1 to yield plasmid 4. A second and third trc promoters were added similarly to generate plasmids 5 and 6. *Synechococcuselongatus* PCC7942 phosphoribulokinase gene *prk* was inserted into the *Bgl*I/*Psh*AI sites of plasmid 2. The promoter *trpR*-P_trp_ was constructed by fusion of the TrpR repressor and the P_trp_ promoter amplified from DH5α genomic DNA. The fused promoter was inserted into the *Fsp*I/*Bgl*I sites of prk-containing vector, yielding the selection plasmid. Overlapping PCR was used to introduce point mutations into the *rbc* or *prk* gene to constructed the corresponding mutants.

### Rubisco expression

Rubisco expression were conducted by 0.2 mmol/L IPTG for 6 h at 22°C. Ten OD_600_ of cells were resuspended in 1 mL buffer A (100 mmol/L HEPES, pH 8.0, 20 mmol/L MgCl_2_, 10 mmol/L KCl, 1 mmol/L EDTA), and sonicated. A 5-μL aliquot each of the supernatant fraction (soluble protein) and the resuspended pellet fraction (insoluble protein) were subjected to SDS-PAGE (12% *w*/*v*) and western blotting with rabbit antibodies against *Synechococcus* sp. PCC7002 Rubisco (0.9 mg/mL, 1:20,000 dilution). Goat anti-rabbit IgG antibody conjugated with alkaline phosphatase (1:7500 dilution) was used as the secondary antibody.

### Library construction

The nonadjacent *rbcL* and *rbcS* genes in the selection plasmid were mutated by a standard error-prone PCR protocol with 0.015 and 0.1 mmol/L Mn^2+^, respectively (Joo et al., 1999). The inner T7-*rbcX* region was amplified by high-fidelity DNA polymerase. Overlap extension of the three fragments was conducted as described in Protein Domain Library Generation by Overlap Extension (PDLGO) (Gratz and Jose, 2008).

### Rubisco purification

A his_6_-tag was fused to the C-terminal of *rbcL* for purification via a nickel-chelating His∙Bind column (Novagen), and the purity was more than 90% as judged by SDS-PAGE. The purified enzymes eluted by the high concentration of imidazole were subjected to buffer exchange with buffer A by YM-3 Microcon device (Millipore). All the purified enzymes were adjusted to at least 500 μg/mL before activity assay.

### Activity assay and kinetic parameters measurement

The carboxylation activity of Rubisco towards CO_2_ was measured spectrophotometrically in a coupled enzyme reaction system (Coba de la Peña et al., 2001; Du et al., 1996; Kane et al., 1998). The activity calculated by this spectrophotometric assay was reported to be consistent with that by the conventional radiometric assay (Lan and Mott, 1991; Lilley and Walker, 1974; Ward and Keys, 1989). The reaction system (200 μL) contained freshly prepared 50 mmol/L HEPES, pH 8.0, 10 mmol/L MgCl_2_, 5 mmol/L KCl, 0.5 mmol/L EDTA, 5 mmol/L ATP, 0.2 mmol/L NADH, 5 mmol/L creatine phosphate, 5 mmol/L DTT, 5 U/mL 3-phosphoglyceric phosphokinase, 5 U/mL glyceraldehyde-3-phosphate dehydrogenase, 20 U/mL creatine phosphokinase, appropriate amounts of enzyme solutions, and various concentrations of NaHCO_3_ and ribulose-1,5-biphosphate (RuBP). Rubisco was pre-activated in 100 mmol/L NaHCO_3_ on ice for 30 min. The reaction was initiated by RuBP addition and immediately monitored at 340 nm for 5 min at 25°C. All the solutions for activity assay were balanced in an anaerobic chamber filled with 100% N_2_ for 30 min before assay. For the specific activity assays, 100 mmol/L NaHCO_3_ and 0.5 mmol/L RuBP were used. For the measurement of kinetic parameters towards CO_2_, 5–30 mmol/L NaHCO_3_ and 0.5 mmol/L RuBP were used. For the kinetic parameters towards RuBP, 100 mmol/L NaHCO_3_ and 0.02–0.2 mmol/L RuBP were used.

## Electronic supplementary material

Below is the link to the electronic supplementary material.

Supplementary material 1 (PDF 68 kb)

## Abbreviations

IPTG: isopropyl β-D-1-thiogalactopyranoside
PRK: phosphoribulokinase
Rubisco: ribulose-1,5-bisphosphate carboxylase/oxygenase
RuBP: ribulose-1,5-biphosphate
