# Supplementary material for: Development of an activity-directed selection system enabled significant improvement of the carboxylation efficiency of Rubisco
Source: Protein Cell. 2014 May 30;5(7):552–62. doi: 10.1007/s13238-014-0072-x (PMC4085280; doi:10.1007/s13238-014-0072-x)
Supplement: Supplementary file 1 — Supplementary material 1 (PDF 68 kb) [file 13238_2014_72_MOESM1_ESM.pdf]

Supplementary material

Development of an activity-directed selection system enabled significant improvement of the carboxylation efficiency of Rubisco

Zhen Cai, Guoxia Liu, Junli Zhang, Yin Li

Table S1. Primers used in this study. The underlined nucleotides indicate the restriction sites as marked in parentheses at the end. The boxed nucleotides represent site mutations.

Name	Sequence
rbc-Nde-for	5’-AAGGTCTCATATGGTTCAGACCAA ATCTGCTGGG-3’ (NdeI)
rbc-Xho-rev	5’-GTGGTGCCTCGAGACCAACAAAACCTTAGTAAC-3’(XhoI)
2ndT7-for	5’-TAAGCTCCTGATCCCGCGAAATTAATACGACTCA-3’
2ndT7-rev	5’-TTTAAACTCCATCTGTATATCTCCTTCTTAAAGTT-3’
3rdT7-for	5’-ATAAACTTTGATCCCGCGAAATTAATACGACTCA-3’
3rdT7-rev	5’-TAAAGTTTTCATCTGTATATCTCCTTCTTAAAGTT-3
trc-for	5’-CTAGATGTTGACAATTAATCATCCGGCTCGTATAATGTGTGGA ATTGTGAGCGGATAACAATTT-3’ (XbaI)
trc-rev	5’-CTAGAAATTGTTATCCGCTCACAATTCCACACATTATACGAGC CGGATGATTAATTGTCAACAT-3’ (XbaI)
prk-for	5’-ATAACTAAGCCATGCCGGCTATGAGCAAGCCAGATCGTGTTG-3’ (BglI)
prk-rev	5’-AATATTTGACTATCGTCACCAGGCTTGAGTATAGCCTGGCTAG ACGCTAGCGGCGACGGG-3’ (PshAI)
trpR-for	5’-CTGAGACTTGCGCATGAAATTACGGGTATTTGTAGGAC-3’ (FspI)
trpR-rev	5’-AAATTTCACGTTCGGCTTATGACGCTTACTACCGCTATT-3’
Ptrp-for	5’-AGTAAGCGTCATAAGCCGAACGTGAAATTTCCTCTCTTG-3’
Ptrp-rev	5’-CGAAGATTGCCGGCATGGCCATTGTCGATACCCTTTTTACGTG-3’ (BglI)

rbcL197-for	5’-TCTTGACTTCACCATGGATGACGAAAACATCAACTCTCAGC-3’
rbcL197-rev	5’-ATGTTTTCGTCATCCATGGTGAAGTCAAGACCACCACGGAGACATT-3’
rbcL341-for	5’-CCGCCACCCTCGGTATCGTAGACCTGATGCGTGAAGACT-3’
rbcL341-rev	5’-CATCAGGTCTACGATACCGAGGGTGGCGGCGCGATCGCCTTCG-3’
prk2021-for	5’-CCGGTTGCGGCATGGCAACCTTCCTAAATCGCCTTGCCGACTTG-3’
prk2021-rev	5’-CGATTTAGGAAGGTTGCCATGCCGCAACCGGAGTCACC-3’
EP-rbcL-for	5’-AAGGAGATATA<u>CATATG</u>GTTCAGACCAAAT-3’ (NdeI)
EP-rbcL-rev	5’-ATCAGGAGCTTAGAGAGTGTCAA-3’
EP-rbcS-for	5’-TCCGATAAAGAGGACATAAATCAATGA-3’
EP-rbcS-rev	5’-GGTGGTGGTG<u>CTCGAG</u>ACCAACAAAACCTTA-3’ (XhoI)
T7-rbcX-for	5’-TTGACACTCTCT AAGCTCCTGAT-3’
T7-rbcX-rev	5’-GATTTATGTCCTCTTTATCGGA-3’
rbcL-his-rev	5’-ATCTTTACCTAGGAGCTTAGTGGTGGTGGTGGTGGTGACCAGAAGAGAGAGTGTCAACGGTATCGAA-3’
rbcL-his-for	5’-CTTCTGGTCACCACCACCACCACCACTAAGCTCCTAGGCCCGCGAAATTAA-3’
